# De novo distal terminal ileum adenocarcinoma mimicking Crohn’s disease and diagnostic challenges in imaging: a case series

**DOI:** 10.1259/bjrcr.20210103

**Published:** 2021-06-24

**Authors:** Dongwhee Lim, Suresh Fernando, Syed Hyder, Shalini Malhotra, Ahmad Miremadi, Mukil Menon

**Affiliations:** 1 Queen Elizabeth Hospital Kings Lynn NHS Foundation Trust, England, United Kingdom; 2 School of Medical Sciences, University of Cambridge, England, United Kingdom; 3 Department of Histopathology, Addenbrooke’s Hospital, Cambridge, United Kingdom

## Abstract

*De novo* small bowel adenocarcinoma (SBA) in the terminal ileum is the least common of the SBA types. However, its highest prevalence is found in the presence of Crohn’s disease (CD). As patients with SBA and CD present with similar symptoms, there is a high chance of misdiagnosing SBA as CD. This can lead to delay in proper diagnosis and can affect prognosis. In this article, we discuss two cases of *de novo* SBA mimicking CD, in the absence of CD, on conventional CT, CT enteroclysis and magnetic resonance imaging (MRI) enteroclysis. Moreover, it underlines the importance of suspecting SBA in cases where there is a lack of response to long-term medical treatment.

## Introduction

The prevalence of small-bowel adenocarcinoma (SBA) is very low, present in just 5% of all cases of gastrointestinal malignancy.^1^ This is due to the alkaline, low-bacterial, high-immunoglobulin A (IgA) and high-hydroxylase environment in the GI tract.^2^ Moreover, *de novo* SBA in the terminal ileum is the least common of the SBA types, found in only 10% of all SBA cases.^3^ Nonetheless, the terminal ileum is the most common site of SBA in patients with Crohn’s disease (CD), with 75% of SBA cases presenting in patients with CD according to a retrospective study conducted from 1993 to 2009^4^ ; notably, this makes the diagnosis of terminal ileal adenocarcinoma in the absence of CD more difficult due to the high possibility of misdiagnosing it as CD. CD is a well-known risk factor for SBA. A 2006 meta-analysis reported a relative risk of 31.2 (95% confidence interval: 15.9–60.9) for small-bowel neoplasm in CD.^5^ In contrast, we herein introduce two cases of *de novo* SBA in the terminal ileum mimicking CD on conventional CT, CT enteroclysis and MRI enteroclysis in the absence of a final diagnosis of CD.

## Case 1

A 64-year-old female presented with a 2-month history of central intermittent non-radiating abdominal pain with vomiting. There was no haematemesis or haematochezia. There was no significant recent weight loss. She had a background history of polymyalgia rheumatica (on long-term steroid), chronic obstructive pulmonary disorder and use of a cardiac pacemaker. She had a smoking history of 45 pack a year but a low degree of alcohol consumption. There was a notable family history of both bowel and breast cancer.

On clinical examination, left iliac fossa tenderness with guarding was noted. Test results of blood drawn at admission showed elevated C-reactive protein (CRP) (80 mg l^−1^) and elevated faecal calprotectin (402 μg/g).

**Figure 1.**
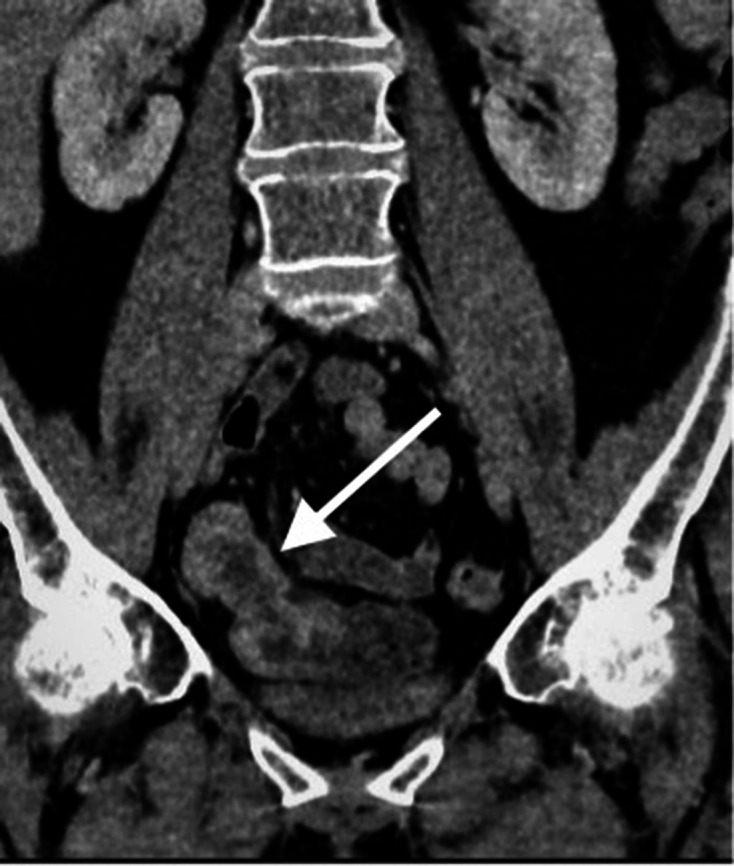
Contrast enhanced CT scan of the abdomen and pelvis in coronal plain demonstrating an approximately 10 cm segment of terminal ileum which shows mural thickening and mild mural hyperenhancement. No locoregional lymphadenopathy or proximal small bowel dilatation.

Initially, gastroscopy and colonoscopy were performed, revealing the existence of normally appearing mucosa. Meanwhile, CT chest–abdomen–pelvis (CAP) imaging showed mild thickening of the distal and terminal ileum throughout a section measuring approximately 10 cm long but no signs of colonic mass or malignancy (Figure 1). As MRI was contraindicated due to the patient’s implanted cardiac pacemaker, CT enteroclysis was performed with volume acquisition following intravenous contrast approximately 12 weeks after the initial CT CAP. This assessment also showed thickening of the mucosa of the terminal ileum, extending to the ileocaecal valve, yet normal appearances of the remainder of the small and large bowels. Clinical and radiological findings suggested possible terminal ileitis with CD.

Based on the image findings and clinical pictures, the patient received 40 mg of methylprednisolone intravenously bd for 2 days and, as her symptoms improved, she was transferred to 40 mg of oral prednisolone 40 mg once daily as a tapering dose. She was discharged with a follow-up plan to be seen by a gastroenterologist in 8–10 weeks’ time as an outpatient.

**Figure 2.**
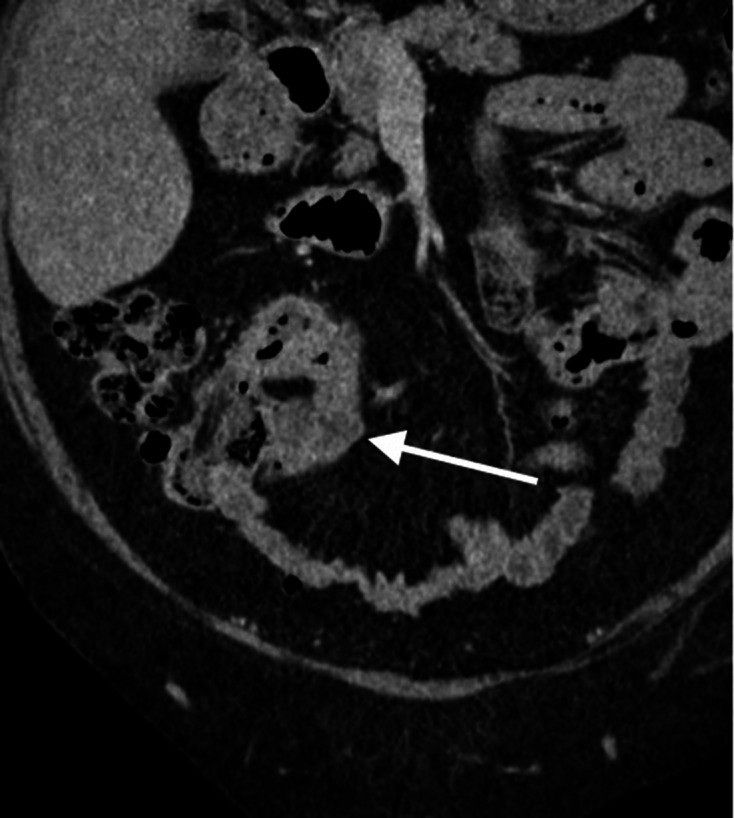
Contrast enhanced CT scan of the abdomen and pelvis in coronal plain demonstrating the previously known segment of terminal ileum which now showing more prominent mural thickening and mild hyper enhancement. No locoregional lymphadenopathy or proximal small bowel dilatation.

3 months later, the patient re-presented with worsening symptoms as her steroid dose was reduced. There was mucus apparent in the stool but no haematochezia. Her white blood cell count (15.45 × 10^9^/L) and CRP level (75 mg l^−1^) were elevated. On clinical examination, her abdomen was soft but mildly tender. An urgent colonoscopy was performed which revealed a terminal ileum tumour. A cold biopsy was taken, which confirmed a moderately differentiated adenocarcinoma. Contrast enhanced CT CAP was performed for staging which showed more prominent mural thickening and mild hyperenhancement of the terminal ileum. However, there was no locoregional lymphadenopathy or proximal small bowel dilatation (Figure 2).

**Figure 3.**
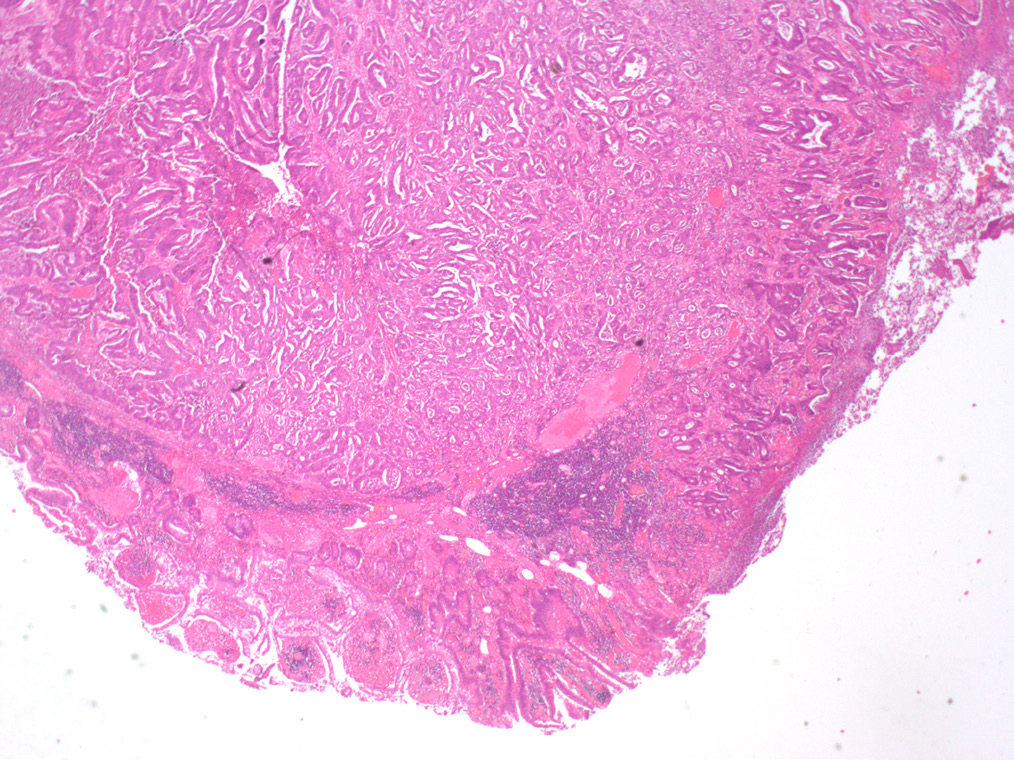
Histopathology sample from the terminal ileum showing moderately differentiated adenocarcinoma in the terminal ileum. Tumour infiltrates 4 mm the beyond the muscularis propria.

At this point, elective laparoscopic right hemicolectomy was pursued. Post-operatively, the histopathology sample of the right hemicolectomy confirmed ulcerated, moderately differentiated adenocarcinoma in the terminal ileum, which had a focal mucinous component (Figure 3). Notably, the latter formed approximately 25% of the tumour, without caecal involvement.

## Case 2

A 60-year-old female presented with a 3-day history of severe generalised abdominal pain but without haematochezia, diarrhoea or significant recent weight loss. She did not have any family history of cancer or inflammatory bowel disease but did have a background history of abdominoplasty for cosmetic reasons as well as hysterectomy and oophorectomy for endometriosis. She was an ex-smoker who quit 12 years prior to the current presentation.

Upon clinical examination, the abdomen was tender in the right and left iliac fossa but per rectum examination findings were unremarkable. Blood biochemistry results at the time of admission involved a mildly increased CRP level of 25 mg l^−1^ but, the rest of the laboratory findings were unremarkable.

**Figure 4.**
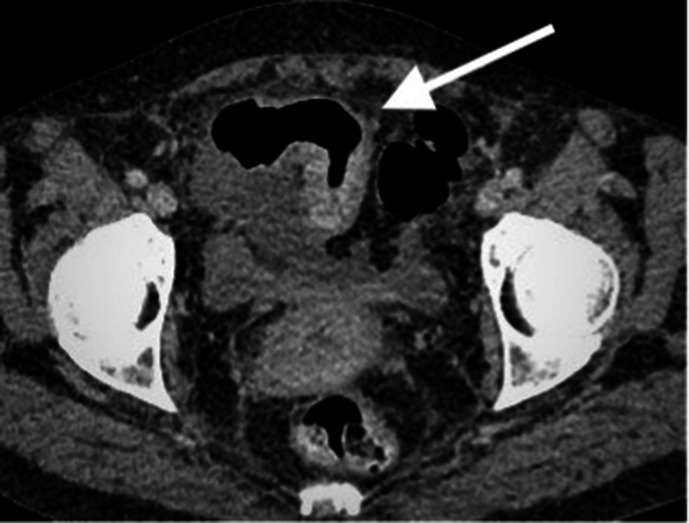
Contrast enhanced CT scan of the abdomen and pelvis in axial plain demonstrating an approximately 8 cm segment of terminal ileum which shows mural thickening and hyperenhancement. No locoregional lymphadenopathy or proximal small bowel dilatation. Small volume free fluid present.

Further investigation with CT CAP imaging revealed an oedematous tubular structure adjacent to the caecum with associated surrounding inflammatory changes, marginally enlarged lymph nodes and free pelvic fluid (Figure 4). It prompted a provisional diagnosis of small-bowel inflammation. The patient continued to improve with intravenous piperacillin/tazobactam. Then she was reviewed by the gastroenterology team and the possibility of CD was considered. The patient was discharged with oral co-amoxiclav and 9 mg of budesonide once daily as a tapering dose along with a follow-up outpatient colonoscopy in 6 weeks. 5 days later, the patient re-admitted with worsening cramping abdominal pain together with nausea and vomiting. An abdominal X-ray showed mildly dilated loops of small bowel. MRI enteroclysis was subsequently performed, revealing a 3.5 cm segment of terminal ileum displaying mural thickening, luminal narrowing and mucosal hyperenhancement, suggesting inflammatory stricture of the terminal ileum. The patient was treated with a course of intravenous methylprednisolone and oral metronidazole and her symptoms again improved.

**Figure 5.**
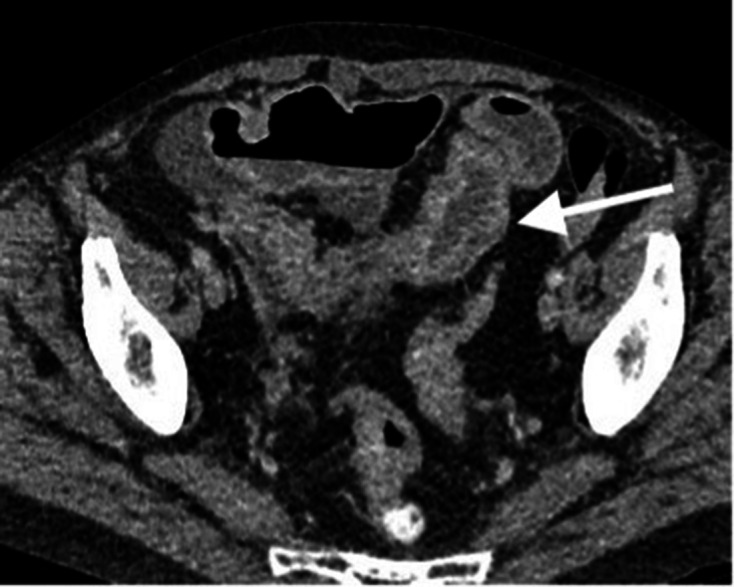
Contrast enhanced CT scan of the abdomen and pelvis in axial plain demonstrating persistent mural thickening of the terminal ileum which shows mural thickening and hyperenhancement. No proximal small bowel dilatation. Small volume free fluid seen on previous study mostly resolved. However, there are few borderline ileocolic lymph nodes and subtle peritoneal nodules (not demonstrated on this image).

**Figure 6.**
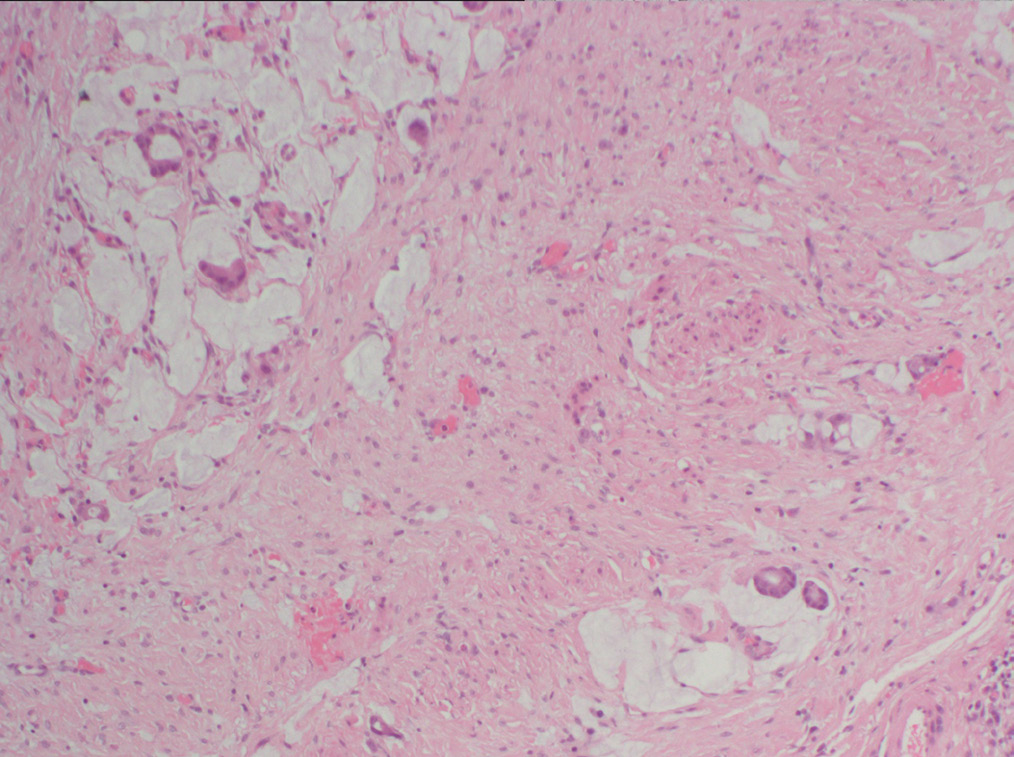
Histopathology sample of peritoneal biopsies showing poorly differentiated adenocarcinoma. There is infiltration of the subserosal fibrous tissue by a population of atypical epithelial cells arranged as single cells and glandular structures with evidence of mucin production.

While awaiting her outpatient colonoscopy visit, she presented again after 1 month, with central, intermittently colicky abdominal pain with vomiting. However, there was no haematochezia. On examination, the patient’s abdomen was distended and mildly tender. Blood biochemistry showed a mildly increased CRP level (39 mg l^−1^) but otherwise normal full blood counts and urea and electrolytes. CT CAP imaging was repeated, showing a segment of possible CD within the terminal ileum and ascending colon, with dilatation of the ileum proximal to the inflammatory stricture. Probable further skip lesions were seen within the jejunum but no intra-abdominal abscess was apparent (Figure 5). An urgent diagnostic laparoscopy was performed. Intraoperatively, extensive peritoneal and small-bowel mesenteric deposits were found. Therefore, a defunctioning loop ileostomy was formed and peritoneal biopsies taken. Biopsy results confirmed poorly differentiated adenocarcinoma of the terminal ileum with multiple peritoneal metastases (Figure 6).

## Discussion

CD is a more common diagnosis than SBA in light of terminal ileal changes on imaging with abdominal pain. The current gold-standard for diagnosing CD is ileocolonoscopy and the conduct of biopsies to examine each colonic segment. CT and MRI are fundamental tools for monitoring small intestinal involvement and penetrating lesions. Nevertheless, the diagnosis of CD should ultimately be comprehensive, taking into account all clinical examinations, radiological findings, blood results and ileocolonoscopy with histology in addition to the patient’s treatment response.

In CD, the common findings on CT and MRI enteroclysis include mural hyperenhancement, wall thickening, ulcers, stenosis and a phenomenon of vasa recta engorgement known as the ‘comb sign’.^6^ Among these, mural hyperenhancement and bowel-wall thickening are the most common findings. The main advantage of MRI is the lack of patient exposure to radiation; moreover, it is superior at detecting fistulas, distinguishing between inflammatory or fibrous changes and strictures. It can also collect information about small bowel motility. Changes in CT and MRI enteroclysis in patients with SBA can look very similar to those with CD. The most common appearance of SBA is luminal narrowing, caused by annular or semi-annular mural thickening. There could be heterogeneous enhancement of the involved small bowel segment. Atypically, there can be polypoid lesions with well-defined margins or ulcerations.^7^ In the early stages of CD or SBA, thickening and hyperenhancement of the mucosa with bowel-wall stenosis may be almost indistinguishable, which could pose a great challenge for diagnosis on cross-sectional imaging.

To understand the prevalence of the presentation of SBA as CD, a literature search was conducted in the PubMed database. Both broad and specific terms such as ‘adenocarcinoma’, ‘carcinoma/cancer’ or ‘tumour/tumor’; ‘small bowel’ or ‘ileum/ileal’; and ‘Crohn’s’ as well as ‘without’, ‘mimicking’, ‘simulating’ or ‘*de novo*’ were included. Secondary search results were also acquired from the references lists of primary search results. The aim of this review was to review SBA cases in the absence of CD. Between 1961 and 2020, a total of seven case reports of terminal ileal adenocarcinoma mimicking CD in patients not previously diagnosed with CD were published.^8–14^ Cases of patients with a longstanding history of CD or with well-controlled CD were eliminated. Due to its rarity, there is a high probability of misdiagnosing adenocarcinoma as CD when the radiological findings show signs of inflammatory strictures. Moreover, clinically, the presentation of SBA and the exacerbation of CD are similar. In a retrospective study of 459 SBA cases between 1970 and 2005, the most common symptoms of SBA were abdominal pain (43%), nausea and vomiting (16%), fatigue and anaemia (15%), gastrointestinal haemorrhage (7%), jaundice (6%) and weight loss (3%).^15^ Bowel obstruction, diarrhoea and fistula are also well-known findings; however, all of these symptoms are also commonly seen in CD. Moreover, symptoms related to malignancy tend to improve with short-term steroid use, which makes the diagnosis of SBA even more complex. The prognosis of cancer majorly depends on successful early detection; however, monitoring the response to steroids and awaiting outpatient colonoscopy delays the diagnosis. In a retrospective study, 67% of cases of CD-related SBA were found incidentally at surgery.^16^

Therefore, clinicians should be more cautious when diagnosing CD in patients with a lack of long-term response to medical treatment such as corticosteroids. Furthermore, the possibility of SBA in the terminal ileum with inflammatory changes on CT or MRI scans should not be overlooked.

## Learning points

- *De novo* small bowel adenocarcinoma (SBA) in the terminal ileum is the least common of the SBA types.
- Changes in CT and MRI enteroclysis in patients with SBA can look very similar to those with CD
- In the early stages of CD or SBA, thickening and hyperenhancement of the mucosa with bowel-wall stenosis can be very difficult to distinguish
- This makes the diagnosis of terminal ileal adenocarcinoma in the absence of CD more difficult due to the high possibility of misdiagnosing it as CD
- Investigations for CD can delay the diagnosis of SBA, so the possibility of SBA should not be overlooked with inflammatory changes in the terminal ileum on CT or MRI, especially with a lack of long-term response to medical treatments
